# A Case of Inversion of the Uterus

**Published:** 1854-02

**Authors:** Lewis Rogers

**Affiliations:** Professor of Materia Medica and Therapeutics in the University of Louisville


### Art. II.—A CASE OF INVERSION OF THE UTERUS.

BY LEWIS ROGERS, M. D., PROFESSOR OF MATERIA MEDICA AND THERAPEUTICS IN THE UNIVERSITY OF LOUISVILLE.

Mrs. Yengst, a German woman, aged forty years, the mother of seven children, all natural births, was taken with the pains of her eighth labor at 9 o'clock, A. M., November 29th, a German midwife in attendance; the labor gradually progressed and was successfully completed, by the birth of a healthy living child, at six o'clock, A. M., November 30th. The placenta and membranes with an umbilical cord twenty-two inches long were expelled very soon after the child; the membranes spontaneously gave way just before delivery, the amount of amniotic liquor not noticed. The labor though rather tedious was not productive of any very marked exhaustion, and the patient was represented to have borne up under the effort, with strength and firmness; the only circumstances worthy of note were moderate hemorrhage throughout its entire progress, coagula of blood passing from time to time, and the coiling of the funis thrice around the child's neck; the hemorrhage was not sufficient to produce material exhaustion, but excited some apprehension on the part of the midwife.

The patient was delivered in the recumbent position, on her back, and the child was neither suddenly nor violently expelled. The placenta was discharged without any assistance from the midwife. Profuse hemorrhage followed the delivery of the placenta, succeeded by faintness and great prostration; the midwife ignorant of the nature of the difficulty and unprepared and unwilling to act, immediately dispatched the husband for me. I saw her at three-quarters past six o'clock, three-quarters of an hour after the completion of the labor. Her condition was then one of extreme prostration; blood had been flowing from her, "like water from a pump," to use her own language; her face was blanched and anxious; hands and feet cold; pulse rapid and feeble; respiration disturbed, with a sense of a want of air; the bed was flooded with blood, though the midwife had been occupied in removing a portion of it to a tub which stood near at hand.

Having administered brandy very freely, as immediate death seemed to be impending, I proceeded to inquire into the nature of the difficulty. Upon placing the hand upon the hypogastrium, instead of finding the uterus hard, globular and contracted as it should be, it passed deep into the cavity of the pelvis and discovered the fundus depressed, and presenting a cup-like form. This state of things at once suggested inversion of the womb, as the probable difficulty, a suggestion confirmed by finding a large, rough, bleeding mass filling up the vagina and protruding slightly beyond the vulva. The diagnosis being obvious, the patient somewhat revived by the brandy, and appreciating the importance of prompt action, I attempted, at once, the reposition of the organ. This was easily accomplished, by embracing the inverted fundus between the thumb and fingers of the right hand, the most dependent portion resting upon the palm, and by gently and gradually pushing it up before them; it was returned with unexpected facility, the os uteri offering scarcely an appreciable resistance. The hand was carried into the cavity of the uterus and retained there sufficiently long to receive assurance of a complete restoration of the organ, and to recognize very decided contraction. Sixty drops of tincture of opium were administered in warm brandy and water, and I left the patient, very shortly after, in a satisfactory condition.

During the day of the 30th, and the following night, Mrs. Y. suffered slightly from after-pains, accompanied by moderate flowing; in the afternoon, nervous and vascular reaction ensued and ran quite high; she complained very much of palpitation of the heart. The after-pains and reaction were treated by opiates; under these and a properly regulated regimen, she gradually improved, presenting scarcely a symptom of uterine inflammation, and was discharged convalescent on the 8th of December. At the present date, 18th December, she has recovered sufficiently to walk about, and to resume her domestic duties, to a limited extent.

This case of inversion of the uterus is reported, not because it presents any point of special interest or peculiarity, in connection with this rare form of accident, but as a contribution to the statistics of the subject, and as the basis of a few remarks in regard to its causes, symptoms, and treatment.

A very relaxed and uncontracted condition of the uterus is universally recognized as essential to the production of inversion ; this state of the organ may be esteemed a requisite predisposing cause, whilst a considerable diversity of agencies may act as exciting causes.

Undue traction of the funis while the placenta is attached to the uterus is one of the most frequently assigned causes of inversion; when the accident occurs, improper management in this respect, on the part of the accoucheur is apt to be suspected. This cause is fully sufficient for the effect in a relaxed condition of the womb. The mere weight of a large and attached placenta, in a similar state of the organ, may also produce the effect, without traction of the funis. This may occur, even in the recumbent position of the patient, but is more likely to take place when she is somewhat elevated in the bed, or when she is delivered in a sitting position, as is common in some parts of the country.

It is not necessary that the placenta be attached to the uterus, in order that traction of the cord may produce inversion. When detached and pressing upon the os uteri, or detained in the vagina, it may excite, by reflex action, the abdominal muscles and diaphragm, to powerful contractions, sufficient not only to extrude the placenta, but also to force down the fundus of the uterus, the lower portion of the organ being, to some extent, a fixed point. This may occur, whether the funis be pulled upon or not, the mere pressure of a foreign body in the uterus and vagina being sufficient to excite the involuntary contractions of the abdominal muscles and diaphragm.

The introduction of the hand into the vagina and cavity of the uterus to search for the placenta will often suffice to produce a straining effort, on the part of the patient, adequate to force down a relaxed fundus. It is even possible that the impression left by the contact of the child with the nerves of the vagina may be reflected, for some length of time after the birth of the child, and sustain the action of the diaphragm and abdominal muscles.

Inversion has followed rude efforts to detach an adherent placenta, by the hand in the uterus; cases of this kind are reported. It is very possible that a similar result might be produced by the common practice of pressing and kneading the uterus, through the walls of the abdomen, for the purpose of soliciting prompt uterine contractions and the speedy expulsion of the placenta, in cases of inertia following delivery. Strong downward pressure directed upon the fundus of a very relaxed uterus would be fully adequate to cause such a dislocation, and the mere probability of such an effect suggests a caution as to this very prevalent mode of practice.

Assuming the erect position has been known to cause inversion, even many days after labor, and the accident has followed the effort connected with alvine evacuation.

A short funis has been assigned as a cause of inversion. A short funis with the placenta attached to the uterus in a state of relaxation would be sufficient to produce inversion, and is in effect the same as intentional traction of a long cord.

Violent expulsion of the child by which it is thrown far from the mother, and the cord thus made forcibly to drag the placenta, either attached or unattached to the uterus, is equivalent to a short cord in its influence upon the uterus, or to undue traction of a cord of the ordinary length. The same is true in reference to the coiling of the cord several times around the neck of the child, a condition which existed in the case of Mrs. Y.

A large quantity of amniotic liquor has been mentioned as contributing to inversion. When a large amount of this fluid is discharged from the uterus simultaneously with the child, the organ is left in a state indisposed to contraction; it is surprised into a condition of inertia. In cases of this kind, profuse homorrhage is very apt to ensue. A physical and vital state of the organ certainly exists here strongly predisposing to the facile action of the other causes assigned; apart from these, it is not easy to comprehend how a large quantity of amniotic water can act as a cause of inversion.

According to the statistics of inversion of the uterus, published by Dr. S. B. Hunt, in the November number of the Buffalo Medical Journal, "the liability to inversion decreases, but not to any marked degree, with the number of children which the woman has borne." This result of Dr. Hunt's analysis is somewhat unexpected, and would scarcely have been anticipated, reasoning from the supposed predisposing cause of inversion. A relaxed and uncontracted state of the uterus seems to be an essential prerequisite to the efficient action of the immediate and determining causes, such as traction of the funis, a short funis, the erect position, abdominal pressure, &c.; it seems almost impossible for the body and fundus of the uterus to be thus displaced, when firmly contracted. Dr. Wm. Hunter, quoted by Dr. Hunt, remarks, "when a uterus is flaccid it is inverted as easily as the finger of a glove, but when it is hard, globular and contracted, it is as difficult to invert as a jack-boot." The question, apart from the testimony of statistics, resolves itself into the further question, whether this necessary flaccidity of the uterus is more apt to supervene after first labors, than in women who have had several children. General experience, which probably cannot be relied on so fully as the results of statistical analysis, would decide in favor of its more likely existence under the latter circumstances. After-pains, due to imperfect contraction of the womb, are very uncommon, with first labors, and become progressively more common, as a general rule, with each succeeding labor. Profuse *post partum* hemorrhage is more prone to occur in a woman who has had many children than after the birth of a first child, for the reason that contraction is not so prompt and vigorous. An analysis of a larger number of cases of inversion would possibly lead to a result somewhat different from that rendered by the interesting researches of Dr. Hunt. First labors are often very protracted, and patients frequently emerge from them very much exhausted; both general and uterine exhaustion may exist, a condition strongly predisposing to the easy operation of those causes supposed to be adequate to induce inversion. If it be true that this accident is more frequent after first and few labors, than in those who have borne many children, this may be the explanation.

Death may ensue very suddenly upon inversion; it may occur before reposition of the organ, though this be promptly attempted, and sometimes very shortly after; in either case it may result from nervous shock or exhaustion, indicated by syncope without hemorrhage, by convulsions without hemorrhage, or there may be profuse hemorrhage superadding its depressing influence to the shock. Hemorrhage alone may be the cause of death, but writers insist, probably too exclusively, upon it as the urgent and immediately dangerous symptom of inversion; it is an important and alarming symptom, but the statistics of Dr. Hunt go to prove that "hemorrhage does not occur to any more fatal extent than does convulsion or syncope." Hemorrhage at once arrests and directs the attention of the intelligent practitioner to the condition of the uterus; examination detects the cause and leads to a prompt interposition of skill. Convulsion and syncope, from the influence of authority, are not so apt to suggest a suspicion of inversion, and thus lead to a prompt examination. In this way, it is possible that many of the chronic cases of inversion are brought about, the true nature of such cases not being detected in time for reposition. It is even possible that life has been lost in many instances, without the cause of death being ascertained. Syncope and convulsions, after labor, though unattended by hemorrhage, may often depend upon unsuspected partial or complete inversion.

Inversion of the uterus demands very immediate attention, not only on account of the threatening symptoms connected with it, but also from the fact that delay increases the difficulty of reposition, in proportion to its duration. All writers insist most emphatically upon the necessity of early attempts at restoration, and such necessity must be obvious. In a very short time, the os uteri contracts and girds the inverted portion so firmly that it cannot be moved. Time is considered so important an element in the successful management of this difficulty that it requires to be measured by minutes; the lapse of an hour may lead to a failure under the most skillful application of the art. Attempts to replace the organ after it has been inverted for several hours and even for many days, have not, however, always been fruitless. Dr. Hunt reports, upon reliable authority, success of the operation, after the expiration of twelve weeks, and at various periods earlier than this. These fortunate cases should not, if possible, be permitted to retard or control the action of the practitioner.

Notwithstanding the necessity which seems almost imperative for early efforts at reposition, regard should be had to the condition of the patient. The nervous shock or the hemorrhage may be such as to render it hazardous to subject the patient, at once, to the further depression necessarily incident to the operation of reposition. If the prostration be very great, some degree of reaction should be established, in the first place, by the liberal use of diffusible stimulants. The same rule of practice, in this regard, which guides the practitioner in the treatment of a case of placenta prævia with profuse bleeding and great prostration, should guide him in a case of inversion. No prudent practitioner would attempt delivery by version of the child, in such a case, until he had rallied his patient by the administration of brandy and laudanum in quantity adapted to the emergency. Though it be dangerous to wait, it is more dangerous to go on, under such circumstances, until some reaction has been restored by artificial stimulation. Surgical operations performed upon patients who have received severe injuries and great shock, immediately after the accident, often determine a fatal result, which might have been averted by a little delay.

While excessive prostration calls for some delay in the operation of reduction, a certain amount of it, with the accompanying relaxation, will materially facilitate success. In the case of Mrs. Y. the ease with which relief was afforded, was doubtless due, in some measure, to the relaxation caused by excessive loss of blood.

According to the analysis of Dr. Hunt, "the placenta is adherent in a large proportion of cases." The question as to the practice proper to be pursued, when this is the case, is an interesting and important one. An attached placenta materially embarrasses the reduction, while its detachment may lead to serious and fatal hemorrhage. On the other hand, if the placenta be returned, its subsequent detachment in the cavity of the uterus may also be attended by very free hemorrhage, and if not very carefully done, may again induce inversion. This latter result has occasionally occurred, and is mentioned among the causes of inversion. It rarely happens that when the placenta is adherent, it is entirely so; its precipitation must necessarily sunder some of its connections; when this is the fact, its complete separation and removal will facilitate and expedite the reduction to such an extent as fully to compensate for the increase of the bleeding surface. Though a larger quantity of blood would certainly be lost from the whole placental surface of the womb than from a portion, in a given period of time, less time and less effort would be required to effect reduction with the placenta detached, and the amound of hemorrhage proportionably diminished. Practitioners of high authority differ in opinion upon this matter. Dewees, Gooch, Burns, Denman and Blundell, very emphatically advise the reduction, if possible, without previously detaching the placenta; Baudeloque, Churchill, Boivin, and Moreau, pursue the other practice. Dr. Ashwell thinks that the danger of hemorrhage is slight in those cases in which the contraction of the cervix hinders the easy reduction of the entire mass; the same contraction ligates the blood-vessels and prevents the loss of much blood. He concludes that "where the protruded organ and its attached placenta do not together make up a large tumor, and where the os does not constrict its upper part, reduction of the whole may be at once attempted; where, however, these favorable conditions do not exist, it will be wiser to ensure reposition by previous separation." The analysis of Dr. Hunt tends to the conclusion that the danger of hemorrhage has been much exaggerated. This gentleman favors separation as an important antecedent step of the operation. His experience and analysis of sixty-seven cases justify the following practical inferences on this point:—that the placenta "when adherent should be removed prior to any attempt to reduce the inversion; such removal of the placenta does not increase, but rather decreases the risk of hemorrhage, while it facilitates reduction; the returning of the placenta, in reducing the inversion, complicates the after treatment, and adds to the danger of hemorrhage, while it retards and renders difficult the reduction."

The case of Mrs. Yengst was unattended by any inflammation of the uterus. The organ was so speedily returned, and subjected to so little manipulation, that this is not remarkable. Cases of inversion seem, however, to enjoy a singular immunity from metritis, even when the reduction has been difficult; this is so far true that Dr. Hunt's analysis leads to the deduction that "there is little danger of metritis occurring after inversion."

